# Pre-treatment nomogram for biochemical control after neoadjuvant androgen deprivation and radical radiotherapy for clinically localised prostate cancer

**DOI:** 10.1038/sj.bjc.6600160

**Published:** 2002-03-04

**Authors:** C C Parker, A R Norman, R A Huddart, A Horwich, D P Dearnaley

**Affiliations:** Academic Department of Radiotherapy and Oncology, The Royal Marsden NHS Trust and Institute of Cancer Research, Downs Road, Sutton, Surrey SM2 5PT, UK; Department of Computing and Information, The Royal Marsden NHS Trust and Institute of Cancer Research, Downs Road, Sutton, Surrey SM2 5PT, UK

**Keywords:** prostate cancer, radiotherapy, neoadjuvant androgen deprivation, prognostic factors, PSA control

## Abstract

Phase III studies have demonstrated the clinical benefit of adding neo-adjuvant androgen deprivation to radical radiotherapy for clinically localised prostate cancer. We have developed a nomogram to describe the probability of PSA control for patients treated in this way. Five hundred and seventeen men with clinically localised prostate cancer were treated with 3–6 months of neo-adjuvant androgen deprivation and radical radiotherapy (64 Gy in 32#) between 1988 and 1998. Median presenting PSA was 20 ng ml^−1^, and 56% of patients had T3/4 disease. Multivariate analysis of pre-treatment factors was performed, and a nomogram developed to describe PSA-failure-free survival probability. At a median follow-up of 44 months, 233 men had developed PSA failure. Presenting PSA, histological grade and clinical T stage were all highly predictive of PSA failure on multivariate analysis. The nomogram score for an individual patient is given by the summation of PSA (<10=0, 10–19=16, 20–49=44, ⩾50=100), grade (Gleason 2–4=0, 5–7=44, 8–10=81) and T stage (T1/2=0, T3/4=35). For a nomogram score of 0, 50, 100 and 150 points the 2 year PSA control rate was 93, 87, 75 and 54%, and the 5 year PSA control rate was 82, 67, 44 and 18%. These results are comparable to those using surgery or higher doses of radical radiotherapy alone. The nomogram illustrates the results of multivariate analysis in a visually-striking way, and facilitates comparisons with other treatment methods.

*British Journal of Cancer* (2002) **86**, 686–691. DOI: 10.1038/sj/bjc/6600160 www.bjcancer.com

© 2002 Cancer Research UK

## 

Radical external beam radiotherapy is a recognised curative treatment strategy for localised prostate cancer, and is given to approximately 30% of men with newly diagnosed prostate cancer in the USA (Mettlin *et al*, 1997). The outcome of radical radiotherapy (RR), in terms of biochemical control, appears to be similar, in comparable patients, to that of alternative treatment modalities, such as radical prostatectomy (Zietman *et al*, 1994; D'Amico *et al*, 1997; Keyser *et al*, 1997; Martinez *et al*, 2000) or brachytherapy (D'Amico *et al*, 1998). However, disease recurrence after radical treatment is common. Long term follow up studies have shown actuarial PSA failure rates of 29% for T1 tumours, 46% for T2a, and as high as 80% for T2b/c, T3 or T4 cancers (Hancock *et al*, 1995; Zietman *et al*, 1995).

The addition of neoadjuvant androgen deprivation (NAD) has been proposed as a means of improving the outcome of radical radiotherapy. As well as reducing the clinical target volume, which, in combination with conformal radiotherapy planning techniques (Shearer *et al*, 1992; Dearnaley *et al*, 1994; Zelefsky *et al*, 1994; Forman *et al*, 1995), may aid safe radiation dose escalation (Yang *et al*, 1995). NAD can also cause additional (or even synergistic) tumour cell kill (Joon *et al*, 1997; Zietman *et al*, 1997). Three randomised trials have shown that the addition of NAD to radical external beam radiotherapy does indeed confer a benefit, in terms of local control, progression-free survival (Pilepich *et al*, 1995; Laverdiere *et al*, 1997; Porter *et al*, 1997), and in the case of RTOG 8610, overall survival (Pilepich *et al*, 2001). These studies include a combined total of just over 400 men treated with NAD and radical radiotherapy. There are few other series of men treated in this way, the largest being a report on 213 patients from Memorial Sloan–Kettering (Zelefsky *et al*, 1998).

One of the problems complicating the clinical study of prostate cancer, is the difficulty in comparing the results of different series. Results may be influenced not just by treatment strategy, but also by case mix, extent of staging procedures, length of follow-up and criteria for determining PSA failure. A recent comprehensive review of the radiotherapy literature found that the published series differed markedly in terms of these factors, making meaningful comparisons difficult (Vicini *et al*, 1998). It might be more useful to compare the outcome of individual subgroups, matched for known prognostic factors, but unfortunately, there is no consensus on the definition of such prognostic subgroups. The use of a nomogram is one way of addressing this difficulty. A nomogram can provide, in an easily accessible form, outcome data in terms of biochemical control, for any given combination of prognostic factors, and, if validated on a separate data set, can also be used to predict the outcome of future patients treated in the same way.

Here, we present the largest series to date of men with clinically localised prostate cancer treated with NAD and radical external beam radiotherapy. These data have been used to produce the first nomogram to describe the probability of PSA control for patients treated in this way.

## PATIENTS AND METHODS

### Patient selection and investigation

Five hundred and seventeen men with histologically proven, clinically localised prostate cancer, treated at the Royal Marsden Hospital between 1988 and 1998, received NAD and radical external beam radiotherapy, and are included in this study. All patients gave informed consent prior to commencement of treatment. Pre-treatment investigations included clinical examination, full blood count, serum creatinine and electrolytes, serum PSA measurement, CT or MRI scan of pelvis, bone scan, P-A chest radiograph, and prostatic sextant biopsies or TURP. Pelvic lymph node sampling was not performed. All biopsy specimens were reviewed at the Royal Marsden Hospital. Men were included if they had T1–4, N0, M0 disease, regardless of presenting PSA, and had no significant co-morbidities, such that they were considered to have a life expectancy of at least 5 years. Radical prostatectomy would have been considered by referring urologists for the minority of patients with suitable cancers, but during the time course of this study radical radiotherapy following NAD has remained the preferred radical option for most patients (Savage *et al*, 1997; Donovan *et al*, 1999). ‘Watchful waiting’ or androgen deprivation alone were used in men for whom radical treatment options were considered inappropriate or who preferred a non-radical treatment approach.

The study period spanned two distinct methods of histological assessment. One hundred and ninety-five cases, examined during the earlier years of the study, were classed as well, moderately or poorly differentiated according to the WHO system. Of the remainder, 299 cases were graded by the Gleason system, while 23 cases had no Gleason score or grading available. For the purposes of the current study, well differentiated tumours were grouped with Gleason scores 2–4, moderately differentiated tumours with Gleason scores 5–7 and poorly differentiated tumours with Gleason scores 8–10. Patient characteristics are listed in Table 1

**Table 1:**
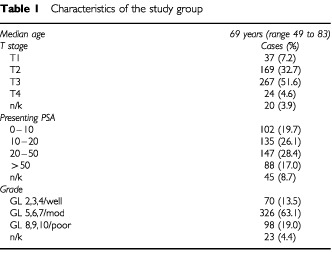
Characteristics of the study group

.

### Treatment protocol

Androgen deprivation was achieved by an initial 3 week course of cyproterone acetate, 100 mg tds orally, together with monthly, subcutaneous leuprorelin 3.75 mg or goserelin 3.6 mg, starting 1 week after cyproterone, and continuing until the completion of radiotherapy. Radical radiotherapy was intended to commence after 3 months of androgen deprivation, but longer courses of initial hormone treatment were allowed for men with bulky local disease, obstructive urinary symptoms, or occasionally when there was uncertainty over the interpretation of initial staging investigations. The median time from starting androgen deprivation to radiotherapy was 111 days (inter-quartile range 97 to 141 days). Adjuvant hormone therapy following radiotherapy was not used. Men were treated supine, with a full bladder. Thirty men were treated in a trial using a pelvic immobilisation device, while for the remainder footstocks alone were used. The planning target volume (PTV) included the prostate gland with a margin of 1 or 1.5 cm, and was localised using CT planning. The seminal vesicles were included in the PTV in men with T3 disease, poorly differentiated (Gleason score 8–10) tumours, or with a presenting PSA of >20 ng ml^−1^. Men were treated with an anterior field and two wedged lateral or postero-lateral fields, using 6–10 MV photons (Dearnaley *et al*, 1999). Conformal shielding was evaluated in a randomised trial until January 1995 (Dearnaley *et al*, 1999), and then adopted during the remaining study period. The planned dose was 64 Gy, specified at the intersection of the treatment beams, delivered in 2 Gy fractions, five times a week, over 6½ weeks. Fourteen patients, treated in the early years of the study, received 60 Gy in 30 fractions.

### Follow-up

Men were seen 6 weeks after starting neoadjuvant hormones, immediately pre-radiotherapy, and on alternate weeks during radiotherapy. They were then followed at 3 to 6 month intervals for 2 years, and annually thereafter. Follow-up included clinical examination and serum PSA measurement, but not routine imaging. Bone scan and CT scan of the abdomen and pelvis was performed if there was clinical suggestion of recurrent disease, or if the PSA was both more than 50% of the presenting PSA, and greater than 10 ng ml^−1^. Continuous, long term androgen deprivation, with either bilateral orchidectomy or LHRH agonist, was started either at the time of PSA failure, or delayed until symptomatic progression, according to the individual preference of the patient and clinician. The Hybritech enzyme immunoassay and the Roche immunometric assay used prior to 1997 provided results to the nearest nanogram per ml, with a lower limit of detection of 1 ng ml^−1^. In January 1997, the Abott AXSYM assay was adopted, with a lower limit of 0.1 ng ml^−1^. Given the limitations of the assays used during the earlier years of the study, we defined biochemical failure as either two consecutive rises in PSA >2 ng ml^−1^, or the commencement of androgen deprivation. The date of PSA failure was taken as the date of the first PSA value >2 ng ml^−1^, or the date of starting androgen deprivation, respectively.

### Statistical methods

Univariate survival analysis of the time to PSA failure was performed using the Kaplan–Meier product limit method (Kaplan and Meier, 1958). Prognostic groups were compared using the log-rank test (Peto and Peto, 1972). Factors with a *P*-value of less than 0.05 were considered significant and were subsequently entered into the multivariate analysis.

The multivariate model was generated using the Cox proportional hazards model (Cox and Oakes, 1984). A *P*-value of 0.05 was used as the criterion to enter or remove variables from the stepwise model. The covariates in the model were organised such that a high relative risk of PSA failure corresponded to a hazard ratio of greater than 1. All variables in the final model were grouped into categories. No continuous variables were used in the final model.

The nomogram was generated by converting the coefficients generated in the model for each prognostic factor (i.e. log of the hazard ratio) onto a linear scale with a maximum based on the maximum coefficient (PSA >50) assuming the value of 100 points. All coefficients for each prognostic group were then plotted relative to this maximum. This allowed easy summation of the risks for any combination of prognostic groups. The total points for a particular patient could then be plotted against the conversion graph to estimate the probability of remaining free from PSA failure for a patient with that particular prognostic group pattern.

The conversion graph was generated from the PSA failure-free survivor function for the baseline covariate pattern. This baseline survivor function was then used to calculate the 1, 2, 3, 4 and 5 year baseline PSA failure-free survival probabilities. The hazards for each prognostic factor from the nomogram were then calculated for all possible covariate patterns and converted to total hazard ratios (relative to the baseline covariate pattern). The PSA failure-free survival probabilities for each of years 1, 2, 3, 4 and 5 were then raised to the power of the total hazard ratios for each covariate pattern to calculate the PSA failure-free survival for each covariate pattern for each year. These were then plotted against the total points corresponding to the relevant covariate pattern from the nomogram. The mean standard error of the PSA failure free survival function for each of the years 1 to 5 was used to estimate the 95% CI of the PSA failure free survival estimate from the nomogram for each year.

## RESULTS

Five hundred and seventeen men with clinically localised prostate cancer were treated with neoadjuvant androgen deprivation and radical radiotherapy. At a median follow-up of 44 months, 233 men developed PSA failure, of whom 20 commenced androgen deprivation on the clinical suspicion of tumour recurrence, without having fulfilled the biochemical criteria for failure. Overall freedom from PSA failure was 68, 56 and 41% at 2, 3 and 5 years, respectively.

Clinical T stage, grade, presenting PSA, and pre-radiation PSA were all highly significant (*P*<0.001) predictors of PSA failure on univariate analysis (Table 2

**Table 2:**
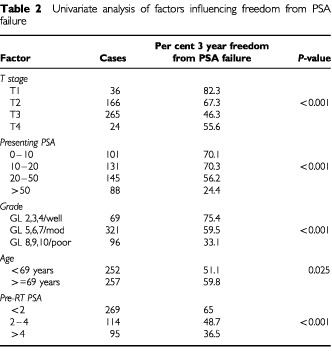
Univariate analysis of factors influencing freedom from PSA failure

). Age was also significant, with older patients faring better than younger, but this effect was less pronounced (*P*=0.025). On multivariate analysis (Table 3

**Table 3:**
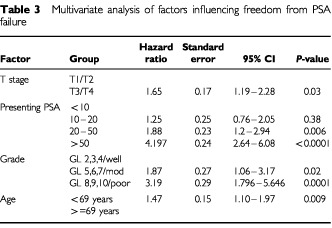
Multivariate analysis of factors influencing freedom from PSA failure

), clinical T stage, grade, presenting PSA and age, but not pre-radiation PSA, were found to be independent prognostic factors for freedom from PSA failure. The results of multivariate analysis of factors predicting for freedom from PSA failure were then displayed in the form of a nomogram (Figures 1

**Figure 1:**
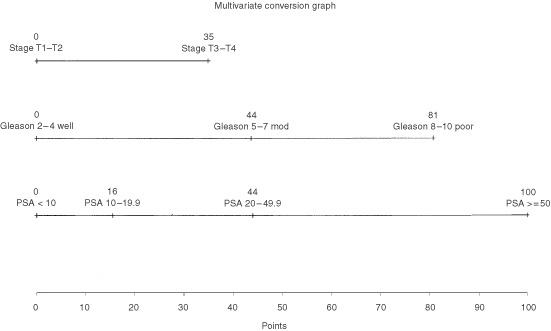
Nomogram: the points for each of the three prognostic factors may be calculated from this nomogram by reading off the x-axis values (e.g. T3 tumours score 35 points). The sum of the points for all three prognostic factors is used in the PSA failure conversion graph (Figure 2

**Figure 2:**
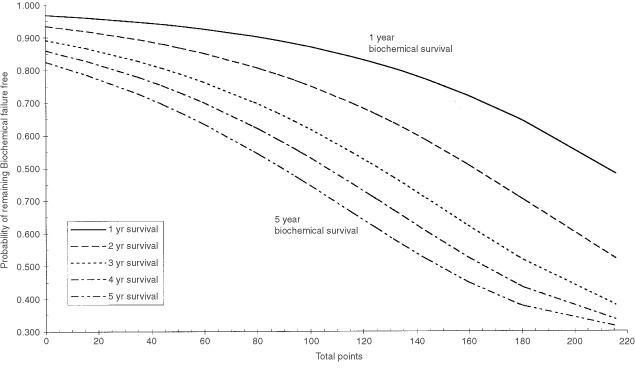
PSA failure conversion graph: The sum of the points from the three prognostic factors (from Figure 1) should be taken on the x-axis and then the percentage of patients free from PSA failure at years 1 to 5 may be read off the y-axis (e.g. for a total points 60 points will have a 5 year freedom from PSA failure rate of 64%).

) to estimate the per cent free from PSA failure at various time points.

and 2), from which the freedom from PSA failure at 1, 2, 3, 4 and 5 years may be identified for any combination of prognostic factors. For example, a man with Gleason score 6, clinical stage T2 prostate cancer with a presenting PSA of 15 ng ml^−1^, has a nomogram score of 60 (44+0+16) points, giving a 5 year freedom from PSA failure of 64%, whereas a man with the same presenting PSA, but with Gleason score 8, clinical stage T3 disease, has a nomogram score of 132 points (81+35+16), and therefore a 5 year freedom from PSA failure of 28%.

Although age was a significant predictor of PSA failure-free survival on multivariate analysis, it was not incorporated into the nomogram in view of the small magnitude of its effect (approximately 4% difference in 5 year freedom from PSA failure, comparing above and below the median age), and in order to facilitate comparisons with other series in which age was not found to be a significant predictor.

The 95% CI of the estimate of freedom from PSA failure was calculated for each of the years 1, 2, 3, 4 and 5 as ±3.6%, ±4.4%, ±4.8%, ±5.1% and ±5.8% respectively. The freedom from PSA failure estimates should therefore be considered to be accurate to within ±5%.

## DISCUSSION

This is the largest study to date of men with clinically localised prostate cancer treated with neoadjuvant androgen deprivation and radical radiotherapy. We have described the outcome in terms of freedom from PSA failure, and have generated a pre-treatment nomogram incorporating the results of multivariate analysis, which describes that outcome for any combination of prognostic factors. The major prognostic contribution of presenting PSA and grade, and the relatively minor contribution of clinical T stage are readily apparent when the data is displayed in this way.

Similar nomograms which have been developed to describe the outcome of men with localised prostate cancer have been catalogued by Ross *et al* (2001). The Memorial Sloan–Kettering nomogram for predicting the outcome of conformal radiotherapy is particularly noteworthy (Kattan *et al*, 2000). Merits of this nomogram are that it was based on a series of over 1000 patients, validated on a separate data set, and that it included both radiation dose and use of NAD as predictor variables. It differs from our nomogram in several important ways. First, the population on which it was based consisted largely of screen-detected early prostate cancer, with a median PSA of 11 ng ml^−1^ (c.f. 20 ng ml^−1^ in the current series), and with 77% (c.f. 45%) having T1/T2 disease. Second, the median radiation dose was 75.6 Gy in 42 fractions, compared with 64 Gy in 32 fractions. Third, the ASTRO consensus definition of PSA failure was used. So, while nomograms may make it easier to compare the results from different centres, they must still be interpreted cautiously, and do not obviate the need for appropriately designed phase III studies.

With these reservations in mind, in our study, the 5 year probability of freedom from PSA failure for a man with a Gleason 6, clinical stage T2 prostate cancer and a presenting PSA of 15 ng ml^−1^ is 64% (95% CI 59–69%). This compares with a probability of 40–72%, depending on RT dose, according to the Memorial Sloan–Kettering nomogram discussed above (Kattan *et al*, 2000), and of 55–75% according to Kattan's pre-operative surgical nomogram (Kattan *et al*, 1998). For a Gleason 8, clinical stage T3 tumour with a PSA of 15 ng ml^−1^, the 5 year probability of freedom from PSA failure in our series is 28% (95% CI 23–33%), compared with approximately 15–53%, depending on RT dose, according to the Memorial Sloan–Kettering nomogram (Kattan *et al*, 2000).

The results of our study confirm, in men treated with neoadjuvant androgen deprivation and radical radiotherapy, the utility of the prognostic factors which are known to be important for men treated either with radiotherapy alone, or with radical prostatectomy, namely, clinical T stage, grade and presenting PSA. In addition, and more surprisingly, age was found to be an independent prognostic factor, with patients younger than 69 years having a significantly poorer outcome than those older than 69 years. A recent comprehensive review of the effect of age on the outcome of localised prostate cancer concluded that age at diagnosis is not a significant determinant of outcome for men treated either with radiation alone or with radical prostatectomy (Parker *et al*, 2001). Our data based on men receiving combined radiation and hormonal therapy generates the hypothesis of a treatment-specific effect of age on outcome. The rate and extent of testosterone recovery after stopping LHRH therapy is age-dependent (Oefelein, 1998), and this could explain the earlier detection of biochemical failure in younger men. RTOG 9202 demonstrated that the duration of testosterone suppression can influence outcome, not just in terms of biochemical control, but also in terms of cause-specific survival (Hanks *et al*, 2000). Although the benefits of neoadjuvant androgen deprivation have been well established in phase III trials (Pilepich *et al*, 1995, 2001; Laverdiere *et al*, 1997; Porter *et al*, 1997), the possibility that the magnitude of this benefit is age-dependent should be tested by subgroup analysis of these studies.

Data concerning the androgen-dependent Shionogi adenocarcinoma in nude mice, which serves as an animal model for prostate cancer, suggests that a good response to neoadjuvant androgen deprivation, defined as a volume reduction of greater than 50%, predicts for improved tumour control following radiotherapy (Zietman *et al*, 1997). This is somewhat analagous to the clinical findings of Zelefsky *et al* (1998), who in their study of 213 men with clinically localised prostate cancer, found that a pre-radiation PSA of <0.5 ng ml^−1^ following neoadjuvant androgen deprivation was an independent favourable prognostic factor. We attempted to address this issue by testing the PSA measured immediately pre-radiotherapy as a possible predictive factor for biochemical control. While the pre-radiotherapy PSA correlates significantly with outcome in terms of freedom from PSA failure on univariate analysis (Table 2), it also correlates with presenting PSA, and is no longer statistically significant on multivariate analysis. The discrepancy between our findings and those of Zelefsky *et al* (1998) could reflect differences in the study populations, or the relative insensitivity of the PSA assay used in the early part of our series.

This study started recruiting in 1988, enabling us to gather the largest series to date of men with clinically localised prostate cancer treated with neoadjuvant androgen deprivation and radical radiotherapy. However, certain aspects of patient management in the earlier part of the study would no longer be regarded as state of the art. First, although Gleason scoring is now widely accepted as the most informative method of grading prostate cancer, it became standard practice at the Royal Marsden Hospital only after the start of this series. We have not re-examined the specimens graded using the previous WHO system, which classified cases into three levels of differentiation, but rather have assumed that these three categories correspond to certain Gleason score groupings. Second, our definition of PSA failure (two consecutive rising PSA levels >2 ng ml^−1^, dated from the first PSA level >2 ng ml^−1^) was constrained by the limited sensitivity of the assays used in the majority of this study. In the future we shall compare with the ASTRO consensus definition of failure (ASTRO, 1997) (three consecutive rises in PSA dated midway between the nadir and first rising level) in patients who have been followed with more sensitive assays. This would show the magnitude of any time lag in definition of time to failure. However it should be remembered that the consensus definition was suggested for patients treated with radiotherapy alone, and the pattern of PSA change after neoadjuvant androgen deprivation and radiotherapy, which depends in part on recovery of testosterone levels, may need further study. Third, our series consists largely of men presenting clinically, rather than with screen detected prostate cancer. The consequent large proportion of men with locally advanced tumours, and high presenting PSA, means that one should be cautious in applying our results to asymptomatic, early cancers. However, it also means that our series is entirely representative of the typical case-mix seen in the UK today.

Recently completed randomised trials in localized prostate cancer have shown benefits for both radiation dose escalation (Pollack *et al*, 2000; Dearnaley *et al*, 2001), and for the use of long-term adjuvant androgen deprivation in addition to NAD (Hanks *et al*, 2000). Those men at greatest risk of local rather than metastatic failure may benefit most from radiation dose escalation, whereas men more liable to distant failure may be better served by adjuvant hormonal treatment. A future report will seek to define these categories for our series of patients. Both of these approaches to intensifying treatment can be expected to carry greater morbidity compared with conventional methods. Do our results permit the identification of a group of men with a sufficiently good outcome that they could be spared the potential extra morbidity associated with these more intensive treatments? For the most favourable group of patients (men with Gleason score 2–4, clinical stage T1/T2 prostate cancers with a presenting PSA of less than 10 ng ml^−1^), 5 year biochemical control was only 82.5%. Even for this group, results could be significantly improved, and they are suitable candidates for phase III trials of treatment intensification.
